# Atypical Manifestations of Cowden Syndrome in Pediatric Patients

**DOI:** 10.3390/diagnostics15121456

**Published:** 2025-06-07

**Authors:** Ekaterina Zelenova, Tatiana Belysheva, Elena Sharapova, Irina Barinova, Alexandra Fedorova, Vera Semenova, Yana Vishnevskaya, Irina Kletskaya, Anna Mitrofanova, Denis Sofronov, Ivan Karasev, Denis Romanov, Timur Valiev, Tatiana Nasedkina

**Affiliations:** 1 N.N. Blokhin National Medical Research Center of Oncology, Ministry of Health of the Russian Federation, 115478 Moscow, Russia; klinderma@bk.ru (T.B.); sharapovae.v@yandex.ru (E.S.); fedorova.ronc@gmail.com (A.F.); sulpiridum@yandex.ru (V.S.); yana_vishn@list.ru (Y.V.); mdsofronov@gmail.com (D.S.); i.karasev@ronc.ru (I.K.); timurvaliev@mail.ru (T.V.); 2 Engelhardt Institute of Molecular Biology, Russian Academy of Sciences, 119991 Moscow, Russia; irina.barinova.98@mail.ru (I.B.); tanased06@rambler.ru (T.N.); 3 Central State Medical Academy of the Administrative, Department of the President of Russia, 121359 Moscow, Russia; 4 Russian Children’s Clinical Hospital, Pirogov Russian National Research Medical University, 117997 Moscow, Russia; ikletskaya@gmail.com; 5 Dmitry Rogachev National Medical Research Center of Pediatric Hematology, Oncology and Immunology, 117198 Moscow, Russia; ms.anna.mitrofanova@yandex.ru; 6 Limited Liability Company, Center of Innovative Medical Technologies, 115191 Moscow, Russia; romanovronc@gmail.com; 7 Federal Network of Expert Oncology Clinics “Euroonco”, 115191 Moscow, Russia

**Keywords:** Cowden syndrome, familial case, *PTEN*, epidermal nevus, diffuse B-cell lymphoma, germ cell tumors, renal cell carcinoma, thyroid cancer

## Abstract

**Background/Objectives:** Cowden syndrome (or *PTEN* hamartoma tumor syndrome) (CS/PHTS) belongs to a group of inherited disorders associated with the development of multiple hamartomas. The clinical presentation of patients may include dysmorphic facial features, macrocephaly, developmental delay, and multiple benign and malignant tumors of various localizations. At the same time, only thyroid cancer is thought to have an increased risk in childhood. Skin lesions in CS/PHTS occur in 90–100% of patients and include multiple tricholemmoma, papilloma, acral keratosis, pigmentation changes, as well as rarer forms like vascular malformations, fibromas, neuromas, melanoma, and basal cell carcinoma. **Methods:** Next-generation sequencing and Sanger sequencing were used to search for *PTEN* genetic variants. A histological and immunohistochemical examination of tumor biopsies and skin lesions was performed. **Results:** A total of 13 patients from six families with CS/PHTS, including 10 children, were described. Seven pediatric patients belonged to families with paternal transmission of the *PTEN* pathogenic variants, while three others were de novo cases. Atypical manifestations in CS/PHTS were diffuse large B-cell lymphoma in one adult, a renal cell carcinoma, three germ cell tumors, and a linear epidermal nevus in pediatric patients. A literature review of the identified pathogenic variants in the *PTEN* gene was performed, assessing their clinical significance and analyzing the traditional and modified diagnostic criteria as applied to the pediatric population. **Conclusions:** Taking into account the low incidence of CS/PHTS, the data presented significantly expand our current understanding of this disease and guide physicians to consider a wider range of possible malignant neoplasms in pediatric patients with CS/PHTS.

## 1. Introduction

Cowden syndrome (CS) is a rare genodermatosis with autosomal dominant inheritance, rather high but incomplete penetrance, and marked variability in clinical presentation [1]. The incidence is thought to be about 1 case per 200,000 people, although this is most probably an underestimate because it is assumed that most patients are undiagnosed [2]. The majority of cases are due to germline pathogenic variants in the *PTEN* gene (CS type 1, OMIM #158350). Rarer types of the syndrome are associated with hypermethylation and abnormal expression of the *KLLN* gene (CS type 4), as well as germline variants in the *SDHB*, *SDHD*, *AKT1*, *PIK3CA*, and *SEC23B* genes [3]. In addition, cases due to somatic mosaicism have been described in the literature [4,5,6].

At the same time, pathogenic variants in the *PTEN* gene can be associated with the development of several inherited diseases of the *PTEN*-associated hamartoma tumor syndrome group. In addition to Cowden syndrome, they include Bannayan-Riley-Ruvalcaba syndrome (BRRS), Lhermitte–Duclos disease, macrocephaly/autism syndrome (OMIM #605309), Proteus-like syndrome, and juvenile polyposis of infancy caused by deletions of the *BMPR1* and *PTEN* genes [7,8]. Therefore, modern authors use the term CS/PHTS when describing patients with Cowden syndrome and a mutation in the *PTEN* gene. Currently, the NCCN diagnostic criteria (Table 1) are used to diagnose CS/PHTS in patients over 18 years of age [9].

The presence of a pathogenic variant in the *PTEN* gene and family history data are taken into account when calculating the criteria (Table 2) [4].

Adult patients with CS/PHTS have an increased risk of malignant neoplasms, primarily breast cancer, endometrial cancer, thyroid cancer, colorectal cancer, renal cell carcinoma, and rarely other carcinomas [10]. The median age of the first tumor detection is 36 years, and the risk of developing second tumors is 8 times higher than in the general population [11]. Sporadic cases of squamous cell carcinoma of the skin and mucosa, ovarian cancer, testicular cancer, prostate adenocarcinoma, hepatocellular carcinoma, and transitional cell carcinoma of the bladder have also been described in patients with CS/PHTS.

As the vast majority of symptoms of CS/PHTS manifest in adulthood, a number of authors have proposed their own criteria for the diagnosis in children. For example, Tan MH et al. 2011 define macrocephaly as an obligatory sign, and the patients must also have at least one of four additional signs: ASD, gastrointestinal polyps, dermatological signs, arteriovenous malformations, and hemangiomas [8]. Bannayan–Riley–Ruvalcaba syndrome considered a phenotypic form of CS in children is characterized by a combination of macrosomia, macrocephaly with frontal bossing, autism spectrum disorders, intellectual disability, multiple hamartomas and skin lesions (pigmented spots and lentiginosis of the penis or vulva, lipomas, and vascular malformations) [12,13]. Among children, malignant tumors are diagnosed quite rarely. In pediatric patients, the risk of developing malignant neoplasia is significantly increased only for thyroid cancer and is 4–12% [14]. At the same time, a few authors highlight the association of germ cell tumors (testicular cancer and ovarian dysgerminoma) with CS/PHTS in the pediatric population [8]. Thus, to date, there is no clear data on the frequency and spectrum of malignant tumors in children with CS/PHTS.

This article analyzes six clinical cases of CS/PHTS, three of which are familial cases with paternal transmission of a pathogenic variant in the *PTEN* gene. The peculiarity of our patients is the presence of malignant tumors in childhood: ovarian germ cell tumors in three girls aged 4, 7, and 8 years; and a renal cell carcinoma in a boy aged 13 years. In one case, the patient’s father developed diffuse large B-cell lymphoma, which is also uncommon in CS/PHTS. Another atypical feature was a linear epidermal nevus in a newborn as the first symptom of CS/PHTS. Based on the data, the applicability of modified criteria for the diagnosis and management of pediatric CS/PHTS patients is discussed.

## 2. Materials and Methods

### 2.1. Patients

A total of 13 patients (10 males and 3 females) aged between 1 month and 50 years at the time of examination and presenting with clinical manifestations characteristic of CS/PHTS were included in the study. All patients were seen by a dermatological oncologist and counseled by a geneticist. Some patients were additionally examined by other specialists (oncologist, neurologist, nephrologist, pulmonologist, cardiologist, and endocrinologist) due to concomitant pathology.

### 2.2. Histological Examination

A histological examination of paraffin-embedded tissues was performed for patients ID1 (linear epidermal nevus), ID12 (germ cell tumor), and ID13 (renal cell carcinoma and sclerosing pneumocytoma). Standard staining with hematoxylin and eosin was used. Immunohistochemistry (IHC) tests were applied for germ cell tumor (patient ID12) using four monoclonal antibodies: SALL4 (Cell Marque, Rocklin, CA, USA), oct3/4 (Cell Marque, Rocklin, CA, USA), pan-cytokeratin C-11 (Abcam, Waltham, MA, USA), CD30 (Cell Marque, Rocklin, CA, USA).

### 2.3. Genetic Testing

Genomic DNA was isolated from blood leukocytes or nevus tissue and skin without lesions (patient ID1) using the QIAmp DNA Mini Kit (Qiagen, Hilden, Germany). NGS sequencing was performed for the probands in all families.

Libraries were prepared with KAPA HyperPrep Kit (Roche, Basel, Switzerland) as described earlier [15]. The libraries were hybridized with coding regions of 35 genes (Table S1), then pooled and sequenced on MiSeq (Illumina, San Diego, CA, USA) (paired-end sequencing, 300 cycles, and 250–300× coverage depth).

Sequencing data were processed and aligned to the reference genome sequence GRCh (hg38). The annotation of nucleotide sequence and variant discovery was performed using the GATK (Genome Analysis Toolkit) algorithm. Interpretation of the identified variants was carried out according to the ACMG guidelines (doi: 10.1038/gim.2015.30) using ClinVar (https://www.ncbi.nlm.nih.gov/clinvar, accessed on 18 September 2024), Varsome (https://varsome.com, accessed on 18 September 2024), or Franklin Genoox (https://franklin.genoox.com, accessed on 18 September 2024) databases. The identified pathogenic or likely pathogenic variants in all cases were verified by Sanger sequencing; primer pairs used are given in Table S2. For the relatives of the probands, only segregation analysis using Sanger sequencing was done.

## 3. Results

Atypical clinical manifestations were identified in six patients with CS/PHTS. Detailed clinical and anamnestic data were collected, and genetic testing of the patients and their available relatives was performed.

### 3.1. Case N°1

**Figure 1:**
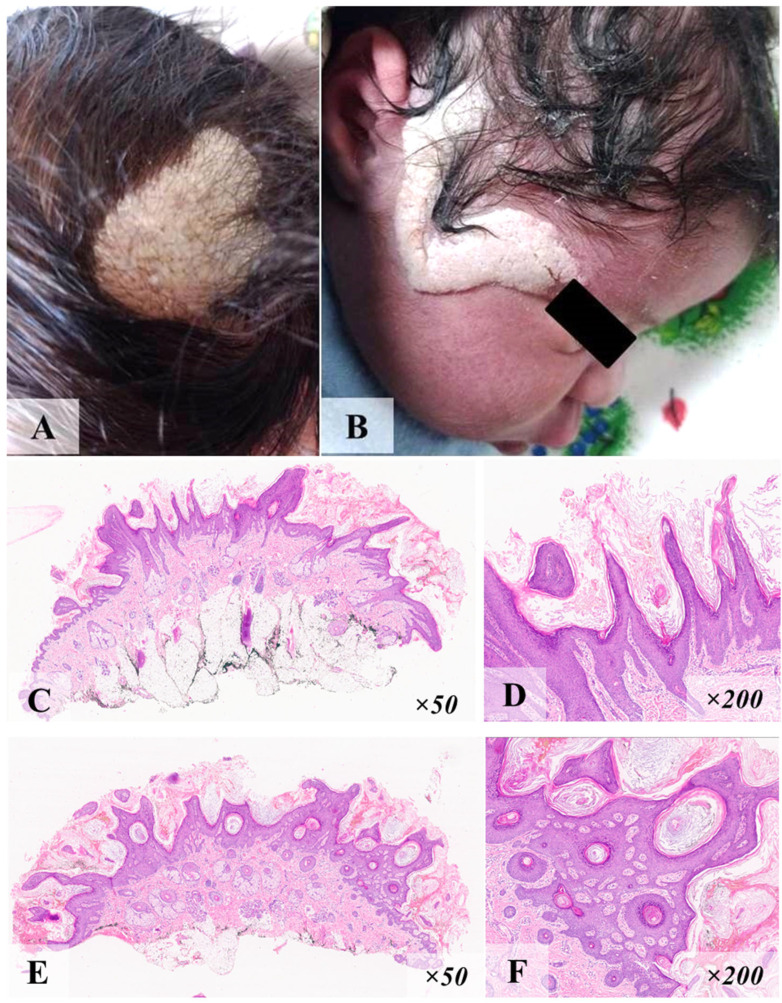
Epidermal nevus of a patient ID1 in the scalp (**A**), and right temporal region (**B**). Histological examination of biopsy (hematoxylin and eosin staining): (**C**,**D**)—papillomatous proliferation of multilayer squamous epithelium of the ‘saw tooth’ type; (**E**,**F**)—superficial layered keratotic masses, the formation of keratocysts of various sizes in the thickness of these outgrowths, and a loose connective tissue stroma at their base.

Patient ID1, a 2-year-old boy, was born to his mother’s sixth pregnancy without peculiarities. At birth, there was marked macrosomia (4330 g/58 cm), and a soft epidermal nevus was apparent on the skin of the scalp and right temporal region. The nevus was a grayish-pink shade with irregular contours and a soft-elastic consistency (Figure 1A,B). The size of the nevus increased in proportion to the child’s height. No other phenotypic manifestations were observed. At 2 months of age, the child was referred to a dermatologist-oncologist, and a nevus biopsy was performed. The morphological picture of the lesion corresponded to a soft epidermal nevus (Figure 1C–F).

A molecular genetic study of the nevus biopsy material was performed by NGS using a panel of skin cancer-associated genes. No pathogenic somatic variants in the *HRAS*, *KRAS*, *NRAS*, or *BRAF* genes were detected; however, in exon 5 of the *PTEN* gene, a variant c.309_312del (p.Phe104ValfsTer8) was found with an alternative allele frequency of 60%. The variant was likely pathogenic according to ACMG criteria (PM4 and PS2) and had not been described earlier. The same variant in the *PTEN* gene in the heterozygous state was detected in the patient’s peripheral venous blood leukocytes, which indicated its germline origin, so the CS/PHTS was diagnosed.

**Figure 2:**
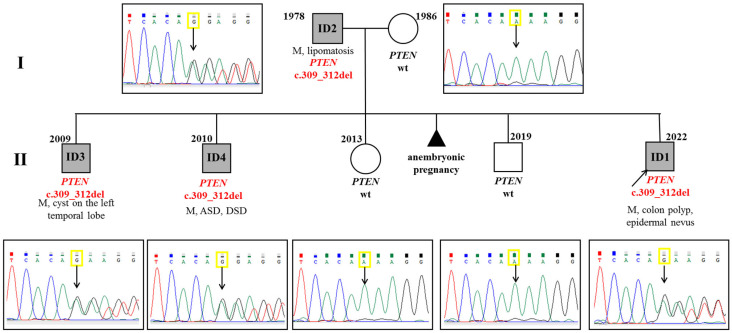
Pedigree of family from case N°1: ASD—autism spectrum disorder, DD—developmental delay, DSD—delayed speech development, M—macrocephaly, wt—wild type. The segregation analysis revealed a mutation in the *PTEN* gene in patients ID1–4, while the proband’s mother, one brother, and a sister did not carry this pathogenic variant.

In a family, the father (ID2) and two brothers (ID3 and ID4) were found to have macrocephaly. One of the brothers (ID3) had postnatal macrosomia (4350 g/53 cm), the other (ID4) had developmental delay and autism, and the father had lipomatosis. The same variant of the *PTEN* gene was found in the father and these two brothers by Sanger sequencing (Figure 2).

At the age of 2 years, the patient ID1 was found to have a colon polyp. Given the small size and single lesion, surgical treatment was not required; however, a colonoscopy was included in the further examination plan.

### 3.2. Case N°2

Patient ID5, a 14-year-old male, was born via cesarean section at 40 weeks due to macrosomia (4230 g/57 cm). In the first year of life, he experienced growth retardation, and a decrease in growth hormone levels in the blood; therefore, the patient received hormone replacement therapy until the age of 1 year. From the age of 11 months, the patient experienced recurrent loss of consciousness, cold sweats, marked weakness, and clonic tremors of the limbs; finally, hypoglycemia (less than 2 mmol/L) of unclear genesis was diagnosed.

The patient was under the dynamic observation of a neurologist for delayed psychomotor and speech development. At 1 year and 5 months of age, rhythm disturbances were noted, namely, continuous recurrent ventricular tachycardia and single- and paired-ventricular extrasystoles. First-degree circulatory insufficiency was diagnosed, and an additional chord of the left ventricle was detected. At the age of 5 years, the papilloma of the left palatine tonsil was surgically removed. During annual follow-up, lipomatosis and colon polyps were revealed at 11 years, and a thyroid nodule (TIRADS-3) was detected at 14 years.

A family history showed that the patient’s younger brother (ID6) had normal height and weight characteristics at birth (3200 g/52 cm). However, there was a delay in speech and psychomotor development (he walked starting at 1 year and 9 months, but does not speak), and at the age of 2 years, this child was diagnosed with infantile cerebral palsy. Furthermore, he had macrocephaly, epicanthus, hypertelorism, cardiac (extra left ventricular chordae, incomplete right bundle-branch block, and resting bradycardia), and orthopedic problems (kyphoscoliosis and hallux valgus of the feet). At the age of 2 years and 10 months, a 4 cm lipoma was found on the skin in the right back region during an annual follow-up.

A comprehensive examination of the patients’ father (ID7) revealed macrocephaly (head circumference 63 cm), multiple papillomas in the axillary and inguinal regions, polyposis of the colon, a lipoma of the jejunum, lymphofollicular hyperplasia of the colon, nodules in both lobes of the thyroid gland, and a vascular malformation in the left cerebellar hemisphere. At the age of 45 years, he was diagnosed with diffuse large B-cell lymphoma, GCB (germinal center B-cell) type of stage IV-B (Ann Arbor classification) involving peripheral, intrathoracic, and intra-abdominal lymph nodes, and lesions of liver, spleen, lungs, and bone marrow. The father underwent polychemotherapy (PCT) according to the RB scheme (rituximab plus bendamustine), and further according to the R-CHOP scheme (rituximab plus cyclophosphamide, doxorubicin, vincristine, and prednisolone) with a positive effect.

**Figure 3:**
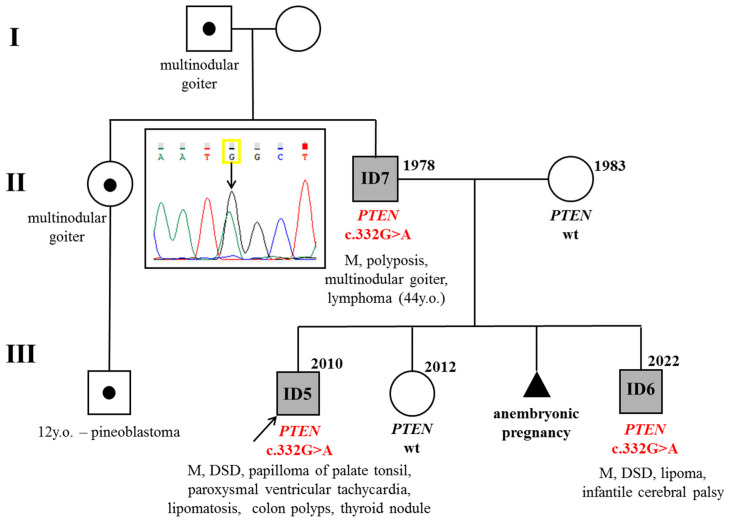
Pedigree from case N°2: M—macrocephaly, DSD—delayed speech development, wt—wild type. Segregation analysis revealed a mutation in patients ID5–7. Mother and sister of the proband are unaffected.

Molecular genetic testing revealed a heterozygous pathogenic variant c.332G>A (p.Trp111Ter) in exon 5 of the *PTEN* gene in proband ID5, his brother ID6, and father ID7 (Figure 3).

### 3.3. Case N°3

**Figure 4:**
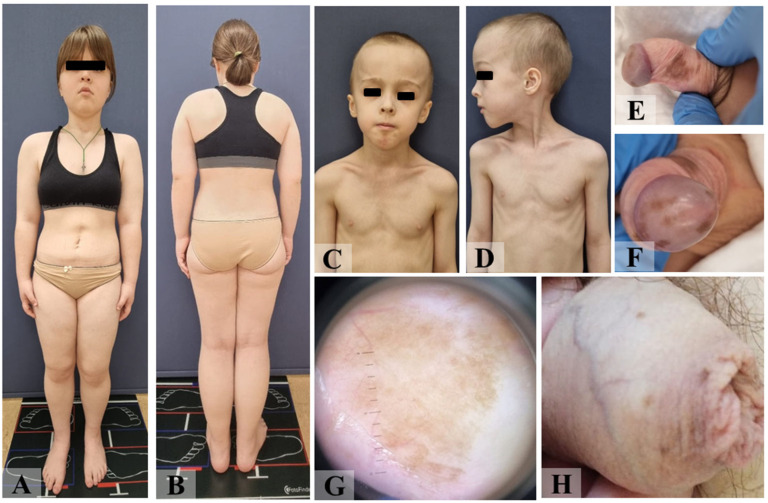
Phenotypic characteristics of the patients from clinical case N°3: proband (**A**,**B**); proband’s brother—bird face, mandibular hypoplasia, microstomia, macrocephaly, high forehead (**C**,**D**), penile lentiginosis (**E**,**F**) with a homogeneous structure revealed by dermatoscopy (**G**); proband’s father—penile lentiginosis (**H**).

Patient ID8, a 15-year-old female (Figure 4A,B) was born with postnatal macrosomia (4350 g/54 cm), but early development was consistent with age. She was seen by a cardiologist until the age of 11 years due to an interventricular septal defect. At the age of 7, a voluminous mass was found in the right ovary and removed laparoscopically, which was defined as a mixed germ cell tumor consisting of mature teratoma (90%) and yolk sac tumor (10%). The patient received three courses of PCT according to the BEP scheme (bleomycin, etoposide, and cisplatin).

At the age of 10 years, a hemithyroidectomy on the left lobe of the thyroid gland was performed due to a follicular adenoma. A year later, the patient underwent thyroidectomy for thyroid cancer in the right lobe, followed by radioactive iodine, and now there are no signs of progression. A more detailed description of the diagnosis and treatment of thyroid cancer in this patient is given by Bricheva E.B. et al., 2024 [16].

**Figure 5:**
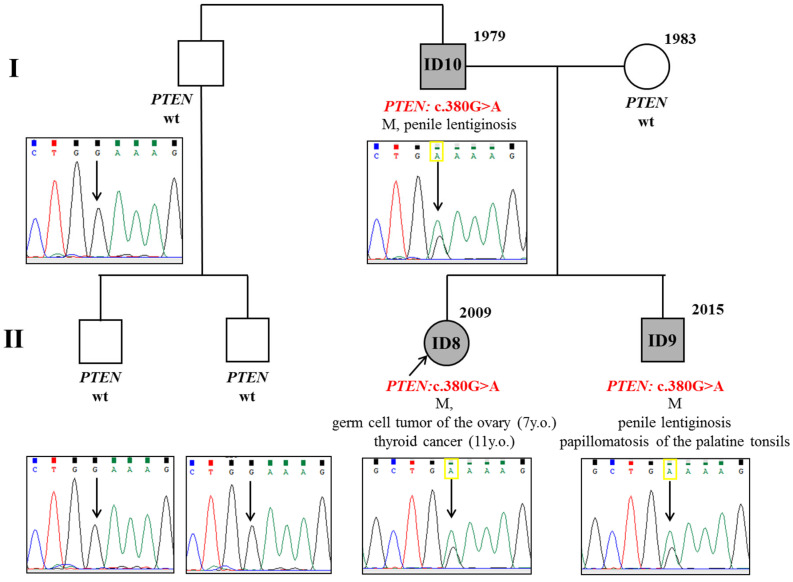
Pedigree of patients from case N°3: M—macrocephaly, wt—wild type. Also, there is the result of segregation analysis: ID8–10 have a mutation, while other family members are unaffected.

Genetic testing revealed a pathogenic variant c.380G>A (p.Gly127Glu) in exon 5 of the *PTEN* gene in the patient’s blood, thus, the diagnosis of CS/PHTS was established. Further pedigree analysis revealed that the patient’s younger brother (ID9) had macrosomia (4310 g/60 cm), macrocephaly, papillomatosis of the palatine tonsils, right-sided cryptorchidism, aplasia of the right testis, and macular pigmentation of the penis from birth (Figure 4C–G), while their father (ID10) has macrocephaly and penile lentiginosis (Figure 4H). The same pathogenic variant c.380G>A was detected in the father and brother, but not in other family members (Figure 5).

### 3.4. Case N°4

Patient ID11, a 14-year-old female, was delivered with vacuum extraction due to macrosomia (4060 g/54 cm). Her mother had a kidney doubling and at the age of 10 years underwent resection of the extra kidney.

The patient grew and developed according to her age; only intracranial hypertension was noted as a neurological symptom. At the age of 4, uterine extirpation with appendages, appendectomy, and resection of the greater omentum was performed because of a malignant germ cell tumor of the ovary, which consisted of embryonal tumor (85% of the tumor tissue), yolk sac tumor (10%), and immature teratoma (5%).

During the four courses of PCT, peritoneal carcinomatosis was noted, and the second surgical intervention aimed at removing metastatic foci was performed. No signs of recurrence were observed over the next 5 years. Since age 11, the patient had been receiving estrogen hormone replacement therapy. At the age of 10 years, multiple thyroid nodules up to 1 cm were detected and continued to grow, so a thyroidectomy was performed another 3 years later, and a multinodular goiter was diagnosed.

**Figure 6:**
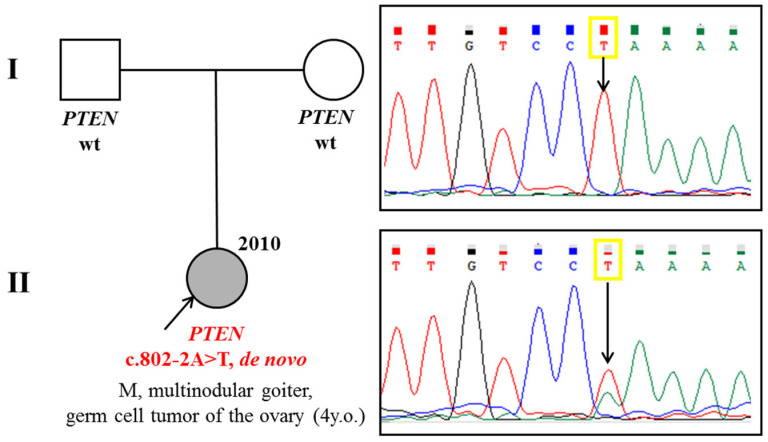
Pedigree and segregation analysis of a patient from clinical case N°4.

Considering multiple tumors, a molecular genetic study was recommended, and a pathogenic variant in the splice site of exon 8 of *PTEN* c.802-2A>T in the heterozygous state was detected. The variant was not found in both parents, so it was considered a *de novo* mutation in this family (Figure 6).

### 3.5. Case N°5

Patient ID12, a 10-year-old female, was born with macrosomia (4294 g/57 cm) and had intracranial hypertension since birth. A fibrolypoma of the supra scapular region appeared at 4 months and was surgically removed at 4 years of age.

**Figure 7:**
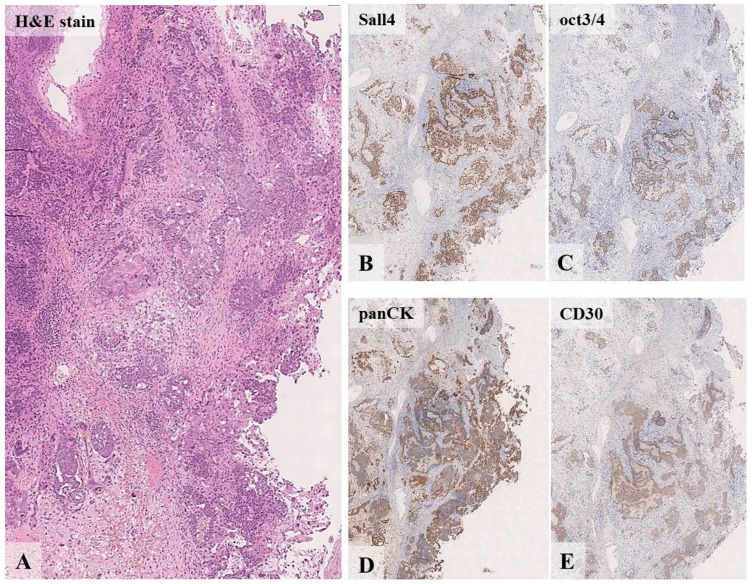
Histological ((**A**), eq. ×200) and immunohistochemical ((**B**–**E**), eq. ×100) examination of the patient’s tumor from clinical case N°5: large cell adenomatoid tumor in fibrous stroma with hemorrhages (**A**); total expression of pangerminative cell marker (**B**); total expression of oct3/4 (**C**); panCK expression by all tumor cells (**D**); CD30 expression by all pathological elements (**E**).

At the age of 8, the proband had macrocephaly, scaphocephaly, and lymphatic malformation on her left thigh. Also, a left ovary tumor was detected on ultrasound. A histological and immunohistochemical examination of the biopsy identified this mass as a germ cell tumor (Figure 7).

**Figure 8:**
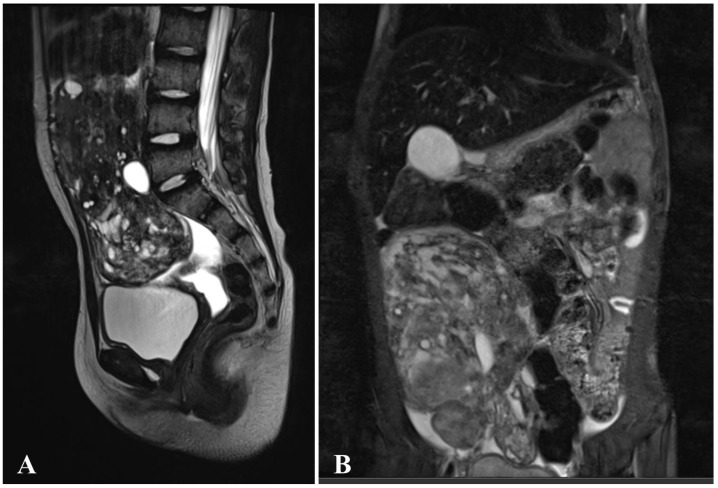
MRI of patient from case N° 5 ((**A**)—T2WI sag, (**B**)—T2 FS cor): massive pelvic tumor with intra-abdominal spread. The tumor has a cystic-solid structure with expansive growth. The upper pole of the tumor reaches the visceral surface of the liver, the lower pole pushes aside the bladder, right kidney, bowel loops, and inferior vena cava.

The patient received PCT according to the MAKEI-96 scheme. After four courses, she underwent an MRI (Figure 8), followed by a salpingo-oophorectomy on the right side. No peritoneal dissemination was detected, the omentum was intact, and the germ cell tumor of mixed structure demonstrated complete therapeutic pathomorphosis.

A molecular genetic study revealed a heterozygous pathogenic variant c.209T>C (p.Leu70Pro) in exon 3 of the *PTEN* gene, confirming the diagnosis of CS/PHTS. The parents refused to undergo segregation analysis.

### 3.6. Case N°6

**Figure 9:**
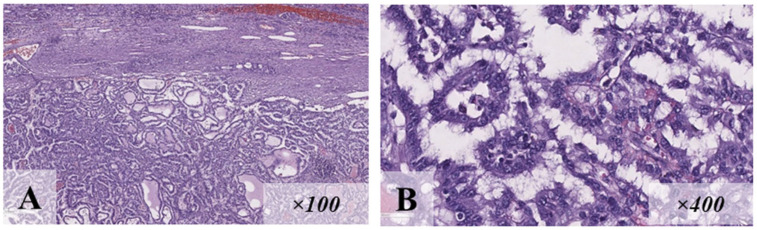
Renal cell carcinoma of patient ID13 (hematoxylin and eosin staining): tumor tissue of papillary structure (**A**); medium-sized cubic cells, nuclei are located basally (**B**).

**Figure 10:**
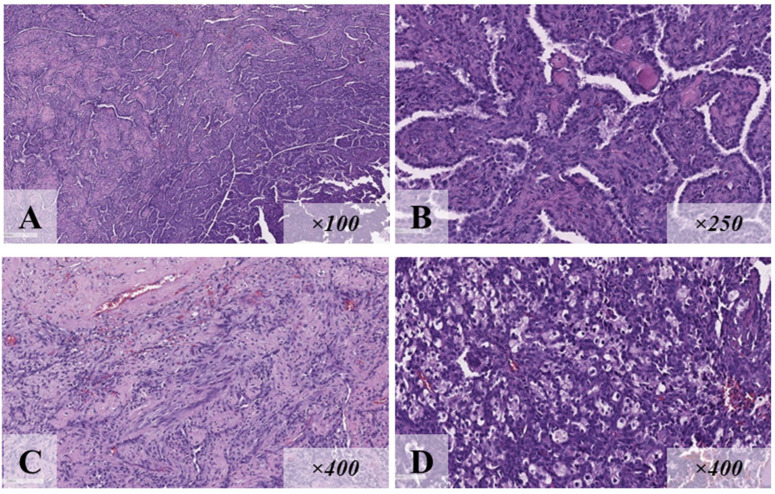
Histological examination (hematoxylin and eosin staining): Sclerosing pneumocytoma of the patient ID13 from clinical case N°6: tumor tissue of papillary and solid structure (**A**); the tumor is represented by superficial cubic cells lining the papillary structures, with rounded basophilic monomorphic nuclei with dispersed chromatin, and rounded cells with eosinophilic cytoplasm, and larger oval nuclei (**B**); there are areas of marked sclerotic changes (**C**) and extensive foci of xanthoma cell aggregations (**D**).

Patient ID13, a 17-year-old male, had pronounced macrosomia (4800 g/58 cm) at birth. He was observed by a neurologist for delayed speech development (he started to speak at the age of 4.5) and intellectual disability. At the age of 2.5 years, he underwent an operation for lymphangioma of the right axillary region. At the age of 13, a renal cell carcinoma of the right kidney developed. The patient underwent nephrectomy (Figure 9), and one month later, a sclerosing pneumocytoma of the left lung was detected (Figure 10). The following phenotypic features were also presented: macrocephaly (head circumference 63 cm), genital lentiginosis, three “café-au-lait” spots on the trunk, gingival hypertrophy, enamel hypoplasia, chest deformity, and scoliosis. At the age of 14, the patient underwent thyroidectomy because of multiple follicular thyroid adenomas. Genetic testing revealed a pathogenic variant c.406T>C (p.Cys136Arg) in exon 5 of the *PTEN* gene in the heterozygous state, which indicated the presence of CS/PHTS.

## 4. Discussion

Thus, the article presents 13 patients with CS/PHTS (Table 3): three familial cases with paternal transmission of the pathogenic variant, and three cases with *de novo* mutations in the *PTEN* gene. The results are summarized in Table 3.

When comparing the phenotype of pediatric patients in our sample with the generally accepted diagnostic criteria [9], we found that only one patient (ID9) could be diagnosed with CS/PHTS because he had a mutation in the *PTEN* gene and three major criteria (macrocephaly, penile lentiginosis, papillomatosis of the palatine tonsils).

Meanwhile, all children in our sample fulfilled the modified pediatric criteria [8,17] and had macrocephaly and, at least, one of the following additional features:

- ASD/expressed developmental delay (ID4, ID5, ID6);
- dermatological features, namely, cyst (ID3), nevus of Jadassohn (ID1), lipoma (ID5, ID6), papillomas on the skin (ID8), fibrolipoma (ID12), and “café-au-lait” spots (ID13);
- anomalies of vascular development (ID13);
- gastrointestinal polyps (ID1, ID5);
- thyroid pathology including multinodular goiter (ID11), thyroid nodule (ID5) follicular adenoma (ID13), and papillary thyroid cancer (ID8);
- germ cell tumor (ID8, ID11, ID12).

Notably, the majority of pediatric patients in our sample had neonatal macrosomia. Thus, the present study emphasizes the need to use modified criteria when assessing the likely phenotypic features of patients with CS/PHTS in pediatric practice.

The spectrum of malignancies in CS/PHTS is quite extensive and includes solid tumors of various localizations in the older age group. Even though hematological malignancies are not typical for patients with CS/PHTS, there are single descriptions of lymphomas in the literature. For example, Galli E et al., 2020, describe Burkitt’s lymphoma in a 57-year-old woman with CS/PHTS who presented with dysplastic cerebellar gangliocytoma at the age of 46 years, papillary thyroid cancer at 47 years, and breast cancer at 51 years [18]. Cavaillé M et al., 2018 described the parent of a patient with CS/PHTS with orbital lymphoma and MALT-lymphoma that developed between 40 and 50 years of age [19]. Another article presented a male patient with CS/PHTS, with B-cell lymphoblastic lymphoma at the age of 7 years and then breast cancer at the age of 31 years [20].

In our case, N°2, a pathogenic variant in the *PTEN* gene was identified in the patient ID7 with different phenotypic manifestations of CS/PHTS and diffuse large B-cell lymphoma diagnosed at the age of 44 years. Thus, the development of non-Hodgkin’s lymphoma cannot be excluded in patients with CS/PHTS, which emphasizes the importance of publishing such cases.

In childhood, malignant tumors in patients with CS/PHTS are extremely rare and are mainly represented by thyroid cancer (TC). In carriers of pathogenic variants in the *PTEN* gene, the lifetime risk of TC is estimated to be 14–38%, with a debut usually in the third decade of life [21]. However, recent studies have demonstrated the possibility of developing TC as early as childhood [14]. The most frequent thyroid pathology in children with CS/PHTS includes nodular goiter and follicular adenomas [1]. Among the patients presented in this study, ID8 manifested TC at 11 years of age, while three other patients had benign thyroid neoplasms since childhood.

The prevalence of renal cell carcinoma (RCC) in carriers of pathogenic variants in the *PTEN* gene is relatively low (1.7 to 4%). Typically, these tumors are unilateral with a debut at the age of 40–50 years, and are predominantly papillary (I and II) or chromophobe histological types [18]. According to studies, the lifetime risk of RCC in patients with CS/PHTS is 34%, with a significant increase after 40 years of age [18,22,23]. At the same time, cases of earlier RCC manifestation have been described in the literature. For example, Kim RH et al., 2020, describe two cases of RCC at a young age in patients with CS/PHTS: a 22-year-old male with macrocephaly and benign thyroid lesions and a 21-year-old female with multiple hamartomas and developmental delay [24].

The development of RCC in childhood has only been presented in an article by Smpokou P, 2015 [25]. The patient described therein had multiple tumors: follicular TC at the age of 7 and then RCC at the age of 11. Our patient ID13 had RCC and a goiter, making these cases similar. Thus, our patient ID13 is the second published case of RCC in children with CS/PTHS. This work is particularly relevant in the context of current clinical guidelines, which recommend screening for RCC in patients with CS/PHTS after the age of 30 years. Consequently, there is no early diagnostics program for adolescents or young adults for RCC, significantly affecting the prognosis of this disease.

Germ cell tumors in CS/PHTS are represented by isolated observations in the literature without a clear correlation with the syndrome. Several cases of immature teratoma [26], ovarian dysgerminoma [27], malignant germ cell tumor of the ovary [28], and seminoma [29] have been described. Our clinical experience, including three cases of malignant germ cell tumors of the ovary in girls aged 4, 7, and 8 years, is the largest series reported in the literature. Such cases call for a revision of clinical guidelines to assess the risk of malignant tumors in patients with CS/PHTS in childhood and the development of a screening program.

Cutaneous manifestations in CS/PHTS are quite diverse [30], but the congenital nevus sebaceous of Jadassohn has been described in only one case with a mosaic variant in the *PTEN* gene [31]. The history of patient ID1 emphasizes the difficulty in the differential diagnosis of this syndrome. Congenital nevus along Blaschko lines is associated with epidermal nevus syndrome (Solomon’s syndrome), which includes Schimmelpenning–Feuerstein–Mims syndrome, phakomatosis pigmentokeratotica, and others. In this regard, molecular genetic studies are an obligatory stage of diagnosis. In the case of our patient, the genetic diagnosis was established in time, which allowed us to take him under dynamic observation and detect colon polyps at the age of 2 years.

Gastrointestinal polyps are found in the majority of patients with CS/PHTS [32], and the lifetime risk of colorectal cancer is 9–16% [33]. This necessitates regular gastro- and colonoscopy; however, according to current standards, such investigations start at the age of 35 years. In this regard, the issue of dynamic follow-up for children with CS/PHTS, especially when polyposis is detected, remains open and requires multicenter studies to elaborate an individual approach to the treatment and management of patients.

Detailed recommendations for children and adolescents with CS/PHTS are presented in the article by Michaela Plamper et al., 2022 [17], but our data demonstrated the necessity of changing the age of beginning follow-up in some positions. For example, the authors suggested starting pelvic tumor screening (yearly testicular/uterine and ovarian ultrasound) at 10 years. Nevertheless, all three girls from our cases (ID8, 11, and ID12) were diagnosed with germ cell tumors at 4, 7, and 8 years. Lung and kidney examinations may also be added to the annual screening plan because of the possibility of pneumocytoma and renal cell carcinoma from puberty, as in the case of our patient ID13.

Establishing this diagnosis in adult patients can be difficult given the differential expression of clinical manifestations and incomplete penetrance, making it important to carefully analyze the pedigree and perform segregation analysis for the next of kin of patients with already verified CS/PHTS. Paternal transmission of pathogenic variants in the *PTEN* gene was noted in three of the six cases we describe. In all cases, the fathers were completely unaware of their diagnosis until the pathogenic variant of the *PTEN* gene was found in their children. At the same time, early diagnosis in these fathers would have decreased the birth of sick offspring using programs of prenatal diagnostics of the fetus or preimplantation diagnosis of embryos in cases of extracorporeal fertilization.

Genetic analysis is important not only for the further planning of pregnancy in patients but also for the prognosis of the disease. Clinical and genetic correlations in patients with CS/PHTS are now widely investigated. For example, in the article by Hendricks LA et al. 2022 [34], the largest number of mutations in patients with CS/PHTS was localized in exon 5 of the *PTEN* gene. Missense variants were associated with earlier disease manifestation, macrocephaly, and developmental delay, while variants leading to premature stop codon formation were more frequently observed in patients with later disease onset, as well as skin, thyroid, and cancer pathologies [34].

**Figure 11:**
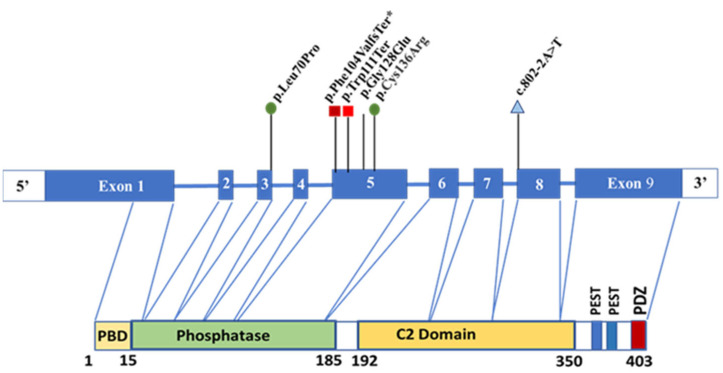
The distribution of pathogenic variants in the *PTEN* gene found in this study.

Among our patients, mutations in exon 5 were also predominant, and developmental delay was observed only in patients with nonsense mutations, coinciding with the data of the above study (Figure 11). However, all patients with malignancies in our sample had missense mutations or a splice site mutation (ID11), and only lymphoma was observed in a father at 44 years of age with a nonsense mutation from clinical case N°2 (ID7). The correlation with the cancer incidence, depending on the type of mutation, is probably not present in the pediatric population.

Additionally, we searched for articles mentioning mutations similar to those identified in our study to better compare phenotypic features. For the variants c.380G>A, c.332G>A, and c.802-2A>T, no clinical description of patients is provided.

The c.406T>C variant (p.Cys136Arg) has been mentioned more than 20 times (including in papers on molecular analyses of the altered function of the encoded protein and large statistical studies). Different clinical manifestations have been described: multiple spinal angiomas and follicular thyroid carcinoma on the background of multinodular goiter [35]; BRRS with arteriovenous malformation [36] and multinodular goiter in 18 years of age, multiple polyps, and ovarian cysts [37]. Our patient ID13 also had vascular pathology in the form of lymphangioma, follicular thyroid adenomas, and renal cell carcinoma. Multiple cancers in the form of thyroid cancer, endometrial cancer, and breast cancer were described in an adult female patient with this mutation [38], and a case of cancer metastasis to cancer in a 75-year-old patient is also known [39]. Cancer in childhood has been described in two patients: an endometrial cancer at 15 years of age and an ovarian tumor at 6 years of age [40]. However, other studies have not found an association with vascular pathology, thyroid pathology, and childhood malignancies in patients with this mutation [41].

The variant c.209T>C (p.Leu70Pro) is quite rare, and only one described case of follicular TC in a 31-year-old male was found in the literature. The patient’s mother had breast cancer at 49 and 53 years of age and endometrial cancer at 63 years of age [42]. Our patient ID12 had no thyroid pathology but was treated for a malignant germ cell tumor of the left ovary at the age of 8 years. Considering the realization of most of the malignancies in CS/PHTS in adulthood, the clinical picture may be complementary; therefore, the patient is under dynamic follow-up with an oncologist.

Thus, even with the same *PTEN* mutation, an extremely wide range of clinical manifestations can be observed, both within the same family and between family cases.

This study has several limitations. Firstly, it is a small sample of CS/PHTS cases with atypical manifestations that were investigated in a single clinical center. Secondly, the NGS gene panel used in this study included only genes associated with cancer, while other genes that could have caused macrosomia were not investigated. Thirdly, only the probands were tested using targeted sequencing, whereas their relatives were examined using direct sequencing to identify a specific genetic defect. Fourth, in some cases, the parents or family members of patients with CS/PHTS were not available or refused to undergo genetic testing to determine the status of the *PTEN* gene.

## 5. Conclusions

The clinical cases described in this article raise the question of a possible expansion of the spectrum of cancers associated with Cowden syndrome by considering pediatric germ cell tumors. The clinical cases also highlight the importance of revising the current guidelines to include patients under 18 years of age in screening programs, not only for thyroid but also for renal cell carcinoma and polyposis. All patients should have their diagnosis verified by molecular genetic methods, as well as undergo a thorough examination for possible clinical manifestations and family history collection to identify all relatives with suspected Cowden syndrome.

## Figures and Tables

**Table 1:** Diagnostic criteria for CS/PHTS.

Major Criteria	Minor Criteria
Macrocephaly (head circumference greater than 58 cm in women and greater than 60 cm in men)	Structural lesions of the thyroid gland (adenoma, adenomatous goiter, etc.)
Follicular carcinoma of the thyroid gland	Thyroid cancer (papillary carcinoma)
Breast cancer	Colorectal cancer
Endometrial cancer	Renal cell carcinoma
Gastrointestinal hamartomas (including ganglioneuromas but excluding hyperplastic polyps; ≥3)	Esophageal glycogen acanthosis (≥3)
Lhermitte–Duclos disease in adults (dysplastic gangliocytoma of the cerebellum)	Intellectual disability (IQ ≤ 75), autism spectrum disorder (ASD)
Macular pigmentation of the glans penis	Testicular lipomatosis
Multiple skin and mucous membrane lesions (≥3): -tricholemmomas; -acral keratosis; -cutaneous mucosal neuromas; -oral papillomas (especially on the gingiva and tongue).	Vascular anomalies
	Lipoma (≥3)


**Table 2:** Application of diagnostic criteria in CS/PHTS.

Family History (at Least One Relative Fulfils the Diagnostic Criteria) and/or the Presence of a Pathogenic Variant in the *PTEN* Gene in the Patient	No Family History, Genetic Status of the Patient Is Unknown/*PTEN*-wt
(1) Any two major criteria with or without minor criteria; OR (2) One major criterion and two minor criteria; OR (3) Three minor criteria.	(1) Three major criteria (one of which is macrocephaly, Lhermitte–Duclos disease, or gastrointestinal malrotation); OR (2) Two major and three minor criteria.

**Table 3:** General characteristics of patients with CS/PHTS (major, minor, and additional criteria) [8,9]. Genomic coordinates are given according to the GRCh38 version.

Patient, Sex, Age	Major Criteria	Minor Criteria	Additional Pediatric Criteria	Atypical Features (Age of Diagnosis, Years)
Case N°1, g.87933068, c.309_312del (p.Phe104ValfsTer8)				
ID1 Male 2y.o.	Macrocephaly	–	Macrosomia (0)	Soft epidermal nevus (0), one hyperplastic polyp of the colon (2)
ID2 Male 47y.o.	Macrocephaly	Lipomatosis		
ID3 Male 16y.o.	Macrocephaly	–	Macrosomia (0), cyst on the skin in the left temporal region	
ID4 Male 15y.o.	Macrocephaly	ASD	DSD	
Case N°2, g.87933091, c.332G>A (p.Trp111Ter)				
ID5 Male 14y.o.	Macrocephaly, one papilloma of the palatine tonsil (5)	Lipomatosis (11) thyroid nodule (14)	DSD, epicanthus, hypertelorism, extra left ventricular chord, resting bradycardia, kyphoscoliosis, hallux valgus	Single hyperplastic polyps of the colon (11)
ID6 Male 3y.o.	Macrocephaly	Lipoma up to 4 cm in size (2y10m)	Macrosomia (0), DSD, paroxysmal ventricular tachycardia	
ID7 Male 47y.o.	Macrocephaly	Multinodular goiter, lipoma of the ileum, vascular malformation in the cerebellum	Lymphofollicular hyperplasia of the colon (18), papillomas in axillary and inguinal areas	Diffuse large B-cell lymphoma GCB type (44)
Case N°3, g.87933139, c.380G>A (p.Gly127Glu), rs398123322				
ID8 Female 15y.o.	Macrocephaly	Papillary thyroid cancer (11)	Macrosomia (0), ventricular septal defect, papillomas in the right axilla, and on the left hand	Germ cell tumor of the right ovary (7)
ID9 Male 10y.o.	Macrocephaly, penile lentiginosis, papillomatosis of the palatine tonsils (10)	–	Macrosomia (0), aplasia of the right testis	
ID10 Male 46y.o.	Macrocephaly, penile lentiginosis	–		
Case N°4, g.87960892, c.802-2A>T, rs587782455				
ID11 Female, 14y.o.	Macrocephaly	Multinodular goiter (10)	Macrosomia (0)	Germ cell tumor of the right ovary (4)
Case N°5, g.87925557, c.209T>C (p.Leu70Pro), rs121909226				
ID12 Female 10y.o.	Macrocephaly	Fibrolipoma of suprascapular region (4 months)	Macrosomia (0), scaphocephaly	Germ cell tumor of the left ovary (8)
Case N°6, g.87933165, c.406T>C (p.Cys136Arg), rs786201044				
ID13 Male 17y.o.	Macrocephaly	Lymphangioma of the right axillary region, follicular adenomas of the thyroid (14)	Macrosomia (0), MR, “café-au-lait” spots, gingival hypertrophy, chest deformation, scoliosis, pulmonary sclerosing pneumocytoma (13)	Papillary renal cell carcinoma (13)

Abbreviations: ASD—autism spectrum disorder; DSD—delayed speech development.

## Data Availability

The original contributions presented in the study are included in the article. Further inquiries can be directed to the corresponding author.

## Supplementary Materials

The following supporting information can be downloaded at https://www.mdpi.com/article/10.3390/diagnostics15121456/s1, Table S1. List of genes in the panel for DNA sequencing; Table S2. Pairs of primers for the mutations in the *PTEN* gene are described in the article.

## Abbreviations

CS/PHTS	Cowden syndrome (or *PTEN* hamartoma tumor syndrome)
CS	Cowden syndrome
ASD	Autism spectrum disorder
BRRS	Bannayan–Riley–Ruvalcaba syndrome
ACMG	American College of Medical Genetics and Genomics
PM	Pathogenic moderate
PS	Pathogenic strong
M	Macrocephaly
DD	Developmental delay
DSD	Delayed speech development
WT	Wild type
PCT	Polychemotherapy
R-CHOP	Rituximab, cyclophosphamide, doxorubicin hydrochloride, vincristine sulfate, prednisone
TIRADS	Thyroid Imaging Reporting and Data System
MRI	Magnetic resonance imaging
NGS	Next-generation sequencing
MALT	Mucosa-associated lymphoid tissue
RCC	Renal-cell carcinoma
TC	Thyroid cancer
