# Supplementary material for: Atypical Manifestations of Cowden Syndrome in Pediatric Patients
Source: Diagnostics (Basel). 2025 Jun 7;15(12):1456. doi: 10.3390/diagnostics15121456 (PMC12192071; doi:10.3390/diagnostics15121456)
Supplement: Supplementary file 1 [file diagnostics-15-01456-s001.zip › Supplementary/Table S1.pdf]

Table S1. List of genes in the panel for DNA sequencing

BRAF	V-RAF MURINE SARCOMA VIRAL ONCOGENE HOMOLOG B1
LRP1B	LOW DENSITY LIPOPROTEIN RECEPTOR-RELATED PROTEIN 1B
TP53	TUMOR PROTEIN p53
TERT	TELOMERASE REVERSE TRANSCRIPTASE
FAT4	FAT ATYPICAL CADHERIN 4
GRIN2A	GLUTAMATE RECEPTOR, IONOTROPIC, N-METHYL-D-ASPARTATE, SUBUNIT 2A
PTPRT	PROTEIN-TYROSINE PHOSPHATASE, RECEPTOR-TYPE, T
PTPRD	PROTEIN-TYROSINE PHOSPHATASE, RECEPTOR-TYPE, D
CDKN2A	CYCLIN-DEPENDENT KINASE INHIBITOR 2A
PREX2	PHOSPHATIDYLINOSITOL 3,4,5-TRISPHOSPHATE-DEPENDENT RAC EXCHANGER 2
NF1	NEUROFIBROMIN 1
ROS1	V-ROS AVIAN UR2 SARCOMA VIRUS ONCOGENE HOMOLOG 1
NRAS	NEUROBLASTOMA RAS VIRAL ONCOGENE HOMOLOG, GTPase
KMT2C	LYSINE-SPECIFIC METHYLTRANSFERASE 2C
NOTCH1	NOTCH RECEPTOR 1
ERBB4	ERB-B2 RECEPTOR TYROSINE KINASE 4
KDR	KINASE INSERT DOMAIN RECEPTOR
ARID2	AT-RICH INTERACTION DOMAIN-CONTAINING PROTEIN 2
FGFR3	FIBROBLAST GROWTH FACTOR RECEPTOR 3
HRAS	HARVEY MURINE SARCOMA VIRUS ONCOGENE, GTPase
ARID1A	AT-RICH INTERACTION DOMAIN-CONTAINING PROTEIN 1A
HDAC9	HISTONE DEACETYLASE 9
PTEN	PHOSPHATASE AND TENSIN HOMOLOG
APC	APC REGULATOR OF WNT SIGNALING PATHWAY
PIK3CA	PHOSPHATIDYLINOSITOL 3-KINASE, CATALYTIC, ALPHA
KIT	KIT PROTOONCOGENE, RECEPTOR TYROSINE KINASE
CTNNB1	CATENIN, BETA-1
TET2	TET METHYLCYTOSINE DIOXYGENASE 2
PDGFRB	PLATELET-DERIVED GROWTH FACTOR RECEPTOR, BETA
ATRX	ATRX CHROMATIN REMODELER
SETD2	SET DOMAIN-CONTAINING PROTEIN 2
TSC2	TSC COMPLEX SUBUNIT 2
SF3B1	SPLICING FACTOR 3B, SUBUNIT 1
TSC1	TSC COMPLEX SUBUNIT 1
MET	HEPATOCYTE GROWTH FACTOR RECEPTOR
GNAQ	GUANINE NUCLEOTIDE-BINDING PROTEIN, Q POLYPEPTIDE
PPP6C	PROTEIN PHOSPHATASE 6, CATALYTIC SUBUNIT
RAC1	RAS-RELATED C3 BOTULINUM TOXIN SUBSTRATE 1
PDGFRA	PLATELET-DERIVED GROWTH FACTOR RECEPTOR, ALPHA
SYK	SPLEEN TYROSINE KINASE
BAP1	BRCA1-ASSOCIATED PROTEIN 1
KRAS	KIRSTEN RAT SARCOMA VIRAL ONCOGENE, GTPase
NF2	NEUROFIBROMIN 2
DDX3X	DEAD-BOX HELICASE 3, X-LINKED
MAP2K2	MITOGEN-ACTIVATED PROTEIN KINASE KINASE 2
GNAS1	GUANINE NUCLEOTIDE-BINDING PROTEIN, ALPHA-STIMULATING ACTIVITY POLYPEPTIDE 1
MITF	MICROPHTHALMIA-ASSOCIATED TRANSCRIPTION FACTOR
MAP2K1	MITOGEN-ACTIVATED PROTEIN KINASE KINASE 1

CCND1	CYCLIN D1
