# Supplementary material for: Atypical Manifestations of Cowden Syndrome in Pediatric Patients
Source: Diagnostics (Basel). 2025 Jun 7;15(12):1456. doi: 10.3390/diagnostics15121456 (PMC12192071; doi:10.3390/diagnostics15121456)
Supplement: Supplementary file 1 [file diagnostics-15-01456-s001.zip › Supplementary/Table S2.pdf]

Table S2. Pairs of primers for the mutations in the *PTEN* gene described in the article

**Pathogenic variant in gene *PTEN***	**Pairs of primers**
Chr10:87933068:c.309_312del (p.Phe104ValfsTer8)	F: GAGGTTATCTTTTTACCACAGTTGC<br>R: TCTAGGGCCTCTTGTGCCTTT
Chr10:87933091:c.332G>A (p.Trp111Ter)	
Chr10:87933139:c.380G>A (p.Gly127Glu)	
Chr10:87933165:c.406T>C (p.Cys136Arg)	
Chr10:87960892:c.802-2A>T	F: ATGAAAATGCAACAGATAACTCAGA<br>R: CTCCTAGAATTAAACACACATCACA
Chr10:87925557:c.209T>C (p.Leu70Pro)	F: ATGAAAATGCAACAGATAACTCAGA<br>R: CTCCTAGAATTAAACACACATCACA
